# Factors Influencing Vaccine Receipt During a 2018 Pediatric Typhoid Conjugate Vaccine Campaign in Navi Mumbai, India

**DOI:** 10.4269/ajtmh.24-0182

**Published:** 2024-09-03

**Authors:** Priyanka Borhade, Christopher LeBoa, Niniya Jayaprasad, Kashmira Date, Pradeep Haldar, Pauline Harvey, Rahul Shimpi, Qian An, Chenhua Zhang, Lily Horng, Kirsten Fagerli, Vijay N. Yewale, Savita Daruwalla, Dhanya Dharmapalan, Jeetendra Gavhane, Shrikrishna Joshi, Rajesh Rai, Varsha Rathod, Keertana Shetty, Divyalatha S. Warrier, Shalini Yadav, Debjit Chakraborty, Sunil Bahl, Arun Katkar, Abhishek Kunwar, Jason R. Andrews, Pankaj Bhatnagar, Shanta Dutta, Stephen P. Luby, Seth A. Hoffman

**Affiliations:** ^1^ World Health Organization–Country Office for India, National Public Health Surveillance Project, New Delhi, India;; ^2^ Division of Infectious Diseases and Geographic Medicine, Department of Medicine, Stanford University School of Medicine, Stanford, California;; ^3^ Global Immunization Division, Center for Global Health, Centers for Disease Control and Prevention, Atlanta, Georgia;; ^4^ Ministry of Health & Family Welfare, Government of India, New Delhi, India;; ^5^ Dr. Yewale Multispecialty Hospital for Children, Navi Mumbai, India;; ^6^ Department of Pediatrics, NMMC General Hospital, Navi Mumbai, India;; ^7^ Department of Pediatrics, MGM New Bombay Hospital, MGM Medical College, Navi Mumbai, India;; ^8^ Dr. Joshi’s Central Clinical Microbiology Laboratory, Navi Mumbai, India;; ^9^ Department of Pediatrics & Neonatology, Dr. D. Y. Patil Medical College and Hospital, Navi Mumbai, India;; ^10^ Rajmata Jijau Hospital, Airoli (NMMC), Navi Mumbai, India;; ^11^ Department of Microbiology, Dr. D. Y. Patil Medical College and Hospital, Navi Mumbai, India;; ^12^ Department of Pediatrics, Mathadi Trust Hospital, Navi Mumbai, India;; ^13^ Department of Microbiology, MGM New Bombay Hospital, Navi Mumbai, India;; ^14^ National Institute of Cholera and Enteric Diseases, Indian Council of Medical Research, Kolkata, India;; ^15^ World Health Organization South-East Asia Regional Office, New Delhi, India

## Abstract

In 2018, the Navi Mumbai Municipal Corporation implemented phase 1 of a public sector typhoid conjugate vaccine campaign in Navi Mumbai, India, targeting all children aged 9 months to 14 years within its administrative boundaries. To assess associations with receipt of vaccine in phase 1, we used generalized estimating equations to calculate estimates of vaccination by child-, household-, and community-level demographics (child education and age; household head education, income, and occupation; community informal settlement percent). Campaign vaccine receipt was most associated with children enrolled in school (odds ratio [OR] = 3.84, 95% CI: 2.18–6.77), the lowest household income tertile when divided into three equal parts (OR = 1.64, 95% CI: 1.43–1.84), and lower community-level socioeconomic status (OR = 1.06, 95% CI: 1.04–1.08 per 10% informal settlement proportion). The campaign was successful in reaching the most underserved populations of its target communities.

In 2019, *Salmonella enterica* serotype Typhi, the causative agent of typhoid fever, was the bacterial pathogen associated with the most deaths in children aged 5 to 14 years in the world.^1^ Globally, *S.* Typhi remains a substantial cause of morbidity and mortality, with an estimated greater than 11 million cases and 116,000 deaths in 2017.^2^ *Salmonella* Typhi had a mortality in the preantibiotic era as high as 12%.^3^ Although effective antibiotics have decreased mortality to less than 1% in most regions,^2^ antimicrobial-resistant *S.* Typhi strains are on the rise^4^^–^^8^ and these strains are associated with increased severity of disease.^9^^–^^11^

In the setting of worsening global rates of antimicrobial-resistant *S.* Typhi, the WHO has prioritized typhoid vaccine delivery in conjunction with water, sanitation, and hygiene interventions.^12^ In 2018, the WHO prequalified a new typhoid conjugate vaccine (TCV) that offers improved immunogenicity and longer duration of protection and that can be administered to children as young as 6 months old.^12^ A trial of TCV in Dhaka, Bangladesh demonstrated 85% protective effectiveness in children ≤15 years old.^13^

Navi Mumbai, India is an urban township adjacent to Mumbai known to have a high burden of typhoid fever.^14^^,^^15^ In 2018, the Navi Mumbai Municipal Corporation (NMMC) implemented a public-sector pediatric TCV campaign (July 14, 2018–August 25, 2018) (for greater detail on campaign decision-making and implementation, please see the study by Date et al.^16^). The campaign endeavored to vaccinate all 9-month-olds to 14-year-olds within NMMC administrative boundaries. The campaign was planned in two phases: phase 1 communities that received TCV during the 2018 campaign and phase 2 communities that were subsequently planned for a follow-up campaign.^16^ Phase 1 and 2 communities were based on Navi Mumbai’s 22 urban health posts and stratified on the proportion of the population living in informal settlements.^16^ The phase 2 communities were planned as an initial comparator for vaccine effectiveness and campaign effectiveness (TCV only privately available at the time).^17^ The phase 2 campaign was indefinitely delayed secondary to the COVID-19 pandemic.

We evaluated the population vaccinated with TCV in areas with (phase 1) and without (phase 2) the campaign. Eligible participants were NMMC residents aged 9 months to 14 years at the start of the campaign (July 2018) who were enrolled from a population-based community assessment survey. This survey was initiated after the campaign and was ongoing between October 2018 and August 2020 in phase 1 and 2 communities. The survey was designed to allow for a sample of 150 to 160 geographically representative clusters every 15 to 16 weeks via a door-to-door questionnaire in a random subset of households across study communities. The questionnaire included information on demographic and socioeconomic characteristics, receipt of typhoid vaccine, history of other childhood immunizations, household wealth, and potential typhoid risk factors. Surveys were collected on password-protected tablets.

The primary definition of “vaccinated” in phase 1 communities was presentation of a TCV vaccination card (from the campaign) or caregiver recall of TCV receipt during the phase 1 campaign, which we refer to as “TCV Vax.” We conducted an additional analysis of TCV recipients in phase 1 and phase 2 communities using a more lenient definition of vaccine card presentation or caregiver recall of TCV vaccination at any time, which we refer to as “TCV Any.”

We performed a generalized estimating equation using the “gee” package in R (version 4.0.4) to calculate population-level estimates of vaccination by child-, household-, and community-level demographics, including child education, age, and gender; household head education, profession, and income; and the percentage of the community composed of informal settlements. Clustering was performed by household study identification code, and the model was run using an independent correlation structure. Reference categories for the model used the lowest income group, children who did not attend school, unemployed household heads, and household heads that achieved a middle school certificate (odds for reference categories were calculated using the reciprocal modeled odds of the category variable with the most substantial modeled odds compared with the reference). Incomplete cases and those who reported not knowing if they received TCV were not included in the model. All analyses were conducted in R version 4.2.2. The exchange rate for US$ to Indian rupee (INR) was 1 US$ = 69.8 INR (converted on August 25, 2018).

The population included in this analysis consisted of 6,414 households (10,878 children) enrolled in the survey (phase 1 communities: 5,919 children, phase 2 communities: 4,959 children). In the phase 1 “TCV Vax” analysis, 305 (5.1%) children were removed due to not knowing vaccination status. In the “TCV Any” analysis, 391 children (6.1%) were removed from phase 1 communities and 572 (11.5%) were removed from phase 2 communities due to missing or nonresponse data. Among households living in phase 1 communities, 56.5% of children were vaccinated with TCV during the campaign and 61.8% of children reported any history of TCV vaccination (campaign or not) (Table 1). Among households in phase 2 communities, 7.9% of children had any reported history of TCV receipt. Campaign-administered vaccine went predominantly to lower-income households, whereas households from the highest income tertile had smaller odds of children being vaccinated by the campaign (odds ratio [OR] = 0.61, 95% CI: 0.53–0.70) (Table 2). Typhoid conjugate vaccine campaign receipt was associated with a 6% increase in odds per 10% increase in the fraction of the community that was composed of informal settlement.

**Table 1:** Characteristics of study population

Characteristics of UHP	Phase 1		Phase 2	Informal settlement (% UHP)
	TCV Vax	TCV Any	TCV Any	
	% (vaccinated/total children)	% (vaccinated/total children)	% (vaccinated/total children)	
Overall	56.5 (3,171/5,614)	61.8 (3,415/5,528)	7.9 (346/4,387)	
Phase 1				
Nerul Sector 48	33.5 (67/200)	48.6 (84/173)		0.0
Nerul 1	39.7 (98/247)	53.4 (264/494)		65.3
Shiravane	46.4 (238/513)	57.7 (581/1,007)		0.3
Juhugaon	48.0 (229/477)	58.8 (446/758)		0.0
Koparkhairane	52.6 (540/1,027)	62.4 (557/892)		0.6
Ghansoli	55.5 (509/917)	62.6 (129/206)		0.6
Digha	60.8 (253/416)	64.3 (270/420)		52.4
Turbhe Store	61.9 (442/714)	67.1 (273/407)		43.5
Airoli	68.5 (307/448)	68.4 (323/472)		13.2
Indiranagar	73.0 (259/355)	68.9 (259/376)		100.0
Chinchpada	76.3 (229/300)	70.9 (229/323)		100.0
Phase 2				
Mahape			2.0 (8/401)	7.9
Nocilnaka			2.8 (11/386)	58.1
Sanpada			3.1 (8/259)	3.5
CBD			4.3 (17/397)	32.2
Ilthanpada			7.5 (48/643)	100.0
Vashigaon			9.9 (57/575)	2.0
Rabada			10.1 (36/356)	0.7
Karave			10.8 (21/195)	0.7
Pawanel			11.8 (43/364)	9.4
Katkaripada			11.9 (58/488)	100.0
Nerul 2			12.1 (39/323)	1.8
Child age (years)				
0 to 5	47.2 (946/2,004)	55.1 (1,089/1,977)	10.4 (166/1,595)	
6 to 10	58.2 (1,030/1,771)	62.2 (1,066/1,715)	4.6 (64/1,382)	
11 to 16	65.0 (1,195/1,839)	68.6 (1,260/1,836)	8.2 (116/1,410)	
Child sex				
Male	55.2 (1,620/2,934)	60.2 (1,747/2,903)	8.2 (186/2,282)	
Female	57.9 (1,551/2,680)	63.5 (1,668/2,625)	7.6 (160/2,105)	
Child education				
Never attended school	43.1 (25/58)	41.7 (25/60)	2.9 (2/68)	
Not of school age	41.1 (450/1,094)	46.3 (506/1,092)	7.5 (63/839)	
Goes to school	60.5 (2,696/4,458)	65.9 (2,884/4,374)	8.1 (281/3,474)	
Household income group (US$ or INR per month)				
US$ 0.0–272.21 (INR 0.00–19,000.00)	61.5 (705/1,149)	63.8 (1,353/2,120)	3.4 (59/1,813)	
US$ 0.00–85.96 (INR 0.00–6,000.00)	61.5 (16/26)	64.4 (29/45)	0.0 (0/69)	
US$ 85.97–272.21 (INR 6,001.00–19,000.00)	61.4 (689/1,123)	63.8 (1,324/2,075)	3.4 (59/1,744)	
US$ 272.21–673.35 (INR 19,000.00–47,000.00)	58.5 (518/885)	61.2 (889/1,453)	7.6 (80/1,058)	
US$ 272.22–458.45 (INR 19,001.00 to 32,000.00)	59.3 (387/653)	62.8 (695/1,106)	5.6 (45/807)	
US$ 458.47–673.35 (INR 32,001.00 to 47,000.00)	56.5 (131/232)	55.9 (194/347)	13.9 (35/251)	
More than US$ 673.35 (INR 47,000.00)	59.5 (132/222)	53.8 (163/303)	20.7 (67/324)	
US$ 673.37–902.58 (INR 47,001.00–63,000.00)	60.4 (84/139)	54.1 (98/181)	11.7 (23/196)	
US$ 902.59–1,805.16 (INR 63,001.00–126,000.00)	54.4 (31/57)	44.6 (33/74)	26.3 (26/99)	
More than US$ 1,805.16 (INR 126,000.00)	65.4 (17/26)	66.7 (32/48)	62.1 (18/29)	
Household head occupation				
Unemployed	56.8 (2,162/3,803)	62.6 (2,324/3,710)	7.8 (216/2,757)	
Trade/unskilled workers	62.3 (314/504)	58.6 (317/541)	2.9 (15/516)	
Skilled worker	60.5 (461/762)	61.2 (479/783)	5.3 (36/681)	
Public official/professional/associate professional	35.4 (125/353)	58.4 (180/308)	25.3 (63/249)	
Household head education				
No formal education	62.1 (380/612)	56.8 (382/673)	2.2 (16/728)	
Primary school certificate (through grade 4)	62.4 (108/173)	59.6 (109/183)	3.8 (6/157)	
Middle school certificate (grade 5–10)	66.1 (1,582/2,394)	50.0 (45/90)	12.3 (10/81)	
High school certificate (grade 11–12)	58.2 (503/865)	65.5 (1,606/2,452)	4.4 (80/1,823)	
Intermediate or post-high school diploma	39.2 (38/97)	61.7 (534/866)	6.6 (41/619)	
Graduate (Bachelor’s degree)	40.4 (427/1,056)	56.4 (524/929)	17.4 (122/703)	
Postgraduate, professional, or honors	31.9 (128/401)	64.6 (210/325)	26.8 (71/191)	

INR = Indian rupees; TCV Any = vaccine card presentation or caregiver recall of typhoid conjugate vaccine (TCV) vaccination at any time; TCV Vax = presentation of a TCV vaccination card (from the campaign) or caregiver recall of TCV receipt during the phase 1 campaign; UHP = urban health post; US$ = U.S. dollars.

**Table 2:** Predictors of vaccination status

Predictors		Phase 1						Phase 2		
		TCV Vax			TCV Any			TCV Any		
		Odds Ratio	CI	*P-*Value	Odds Ratio	CI	*P-*Value	Odds Ratio	CI	*P-*Value
(Intercept)		0.49	0.23–1.03	0.107	0.60	0.29–1.25	0.245	0.1	0.02–0.45	0.01
Proportion neighborhood is informal settlement (10% interval)		1.06	1.04–1.08	<0.001	1.04	1.02–1.06	0.002	0.93	0.89–0.98	0.036
Child age (1-year interval)		0.98	0.97–1.00	0.065	0.96	0.95–0.98	0.002	0.89	0.86–0.92	<0.001
Sex (female = 1)		1.12	1.00–1.26	0.039	1.13	0.99–1.29	0.073	0.88	0.69–1.11	0.285
Child education	Never attended school	Reference			Reference			Reference		
	Not of school age	1.20	0.67–2.15	0.597	1.03	0.58–1.84	0.922	0.49	0.11–2.16	0.307
	Goes to school	3.84	2.18–6.77	<0.001	3.40	1.95–5.95	<0.001	1.16	0.27–4.97	0.828
Household income group (US$ or INR per month)	US$ 0.0–272.21 (INR 0.00–19,000.00)	Reference			Reference			Reference		
	US$ 272.21–673.35 (INR 19,000.00–47,000.00)	0.84	0.73–0.98	0.103	0.89	0.76–1.04	0.261	1.44	0.99–2.09	0.177
	More than US$ 673.35 (INR 47,000.00)	0.61	0.53–0.70	<0.001	0.61	0.47–0.81	0.018	2.28	1.63–3.21	0.001
Household head occupation	Unemployed	Reference			Reference			Reference		
	Trade/unskilled worker	0.91	0.74–1.12	0.526	0.76	0.62–0.94	0.081	0.77	0.43–1.35	0.553
	Skilled worker	1.05	0.89–1.24	0.68	0.88	0.72–1.06	0.329	0.79	0.54–1.15	0.307
	Public official/professional/associate professional	0.80	0.62–1.03	0.18	0.88	0.63–1.24	0.567	1.56	1.08–2.24	0.039
Household head education	No formal education	0.81	0.67–0.99	0.16	0.74	0.60–0.91	0.05	0.6	0.34–1.04	0.19
	Primary school certificate (through grade 4)	0.85	0.61–1.19	0.49	0.74	0.52–1.04	0.189	1.02	0.43–2.42	0.972
	Middle school certificate (grade 5–10)	Reference			Reference			Reference		
	High school certificate (grade 11–12)	0.80	0.68–0.95	0.047	0.87	0.71–1.06	0.296	1.29	0.87–1.93	0.338
	Intermediate or post-high school diploma	0.40	0.26–0.61	<0.001	0.64	0.37–1.10	0.201	2.06	0.99–4.30	0.096
	Graduate (bachelor’s degree)	0.43	0.37–0.51	<0.001	0.72	0.58–0.90	0.026	2.89	2.09–3.99	0.001
	Postgraduate, professional, or honors	0.33	0.26–0.42	<0.001	1.44	0.99–2.11	0.146	4.08	2.71–6.13	<0.001

INR = Indian rupees; TCV Any = vaccine card presentation or caregiver recall of typhoid conjugate vaccine (TCV) vaccination at any time; TCV Vax = presentation of a TCV vaccination card (from the campaign) or caregiver recall of TCV receipt during the phase 1 campaign; US$ = U.S. dollars.

Phase 1 children enrolled in school were more likely to report TCV receipt than those who were not enrolled in school (OR = 3.84, 95% CI: 2.18–6.77). Children from phase 1 households in which the household head reported increasing education-level attainment, compared with the attainment of a middle school certificate, were less likely to report TCV receipt (Table 2).

Among phase 2 households (did not receive the campaign), child TCV receipt was more likely in educated and higher-income families. Households that made more than US$673.35/month (INR 47,000.00; highest income tertile) had a 2.28-fold (95% CI: 1.63–3.21) greater odds of TCV receipt than households that made less than US$272.21/month (INR 19,000.00; lowest income tertile). Children from phase 2 households in which the household head reported a bachelor’s degree (OR = 2.89, 95% CI: 2.09–3.99) or postgraduate/professional degree (OR = 4.08, 95% CI: 2.71–6.13) were more likely to report TCV receipt.

Childhood school enrollment and lower household- and community-level socioeconomic statuses were most associated with TCV receipt via the phase 1 campaign. Among phase 2 communities, wealthier households were more likely to report TCV receipt. Utilizing phase 2 communities as a real-world counterfactual to the phase 1 public-sector TCV campaign, our data portray lower socioeconomic status households and communities as overwhelmingly benefited by the campaign. The 2018 NMMC pediatric TCV campaign, which targeted children living in phase 1 communities, effectively reached children living in households who were disproportionately at increased risk for typhoid fever secondary to low reported household income.^18^^,^^19^ The campaign demonstrated good protection against typhoid fever at the community level in a separate analysis.^17^

This analysis is limited by reliance on self-reported TCV receipt. Participants more aware of the campaign or those with better vaccination records may be overrepresented. The systematic, population-based community assessment survey endeavored to enroll a sample population representative of the study population to reduce bias associated with self-reported TCV receipt. Incomplete cases and those who reported not knowing if they received TCV were not included in our analysis, which may bias our results if responses were due to systematic rather than random causes; however, these represented only a small fraction of our response data. Our major findings remain consistent after application of a stricter definition requiring presentation of a TCV vaccination card. This definition increased the percent vaccinated to 71.4% (765/1,071), reduced the number of children included in the “TCV Vax” phase 1 analysis (from 5,614 to 1,071), led to wider odds ratio 95% CIs, and resulted in increases in missing demographic response data. Among participants included in this analysis, the phase 1 campaign’s TCV coverage was nearly 57%, in comparison with the previously reported administrative campaign coverage estimate of 71%.^16^ This discrepancy could be due to incomplete coverage of the community survey, as well as our study’s reliance on self-reported TCV receipt, as missing data alone cannot account for the 14% difference in coverage estimates. However, because 71% was an administrative estimate, the initial estimate of children in NMMC-administered Navi Mumbai may have been smaller than the true count and may also speak to the effectiveness of the campaign in seeking out all eligible children in Navi Mumbai.

Our findings support the importance the NMMC has placed on TCV vaccination campaigns to successfully reach impoverished communities, especially as part of the potential introduction of TCV into regional and national vaccination programs. Without a public program designed to reach low-income households, wealthier populations may be more likely to benefit from the availability of TCV even when their income status is already known to be protective against typhoid incidence.^18^^,^^19^ Despite the achievement of the TCV introduction in Navi Mumbai, a large proportion of the target population was not reached by this public sector pediatric TCV campaign; even this successful program will require additional pathways of access to reach optimal levels of vaccine coverage.
